# The Ophthalmoscope and Its Use in the Diagnosis of the Diseases of the Deeper Parts of the Eye

**Published:** 1860-09

**Authors:** J. W. Hulke

**Affiliations:** Assistant Surgeon to the Royal London Ophthalmic Hospital, Moorfields, and to King’s College Hospital, London


*The Ophthalmoscope and its use in the Diagnosis of the Diseases of the deeper parts of the Eye.* By J. W. HULKE, F. R. C. S., Assistant Surgeon to the Royal London Ophthalmic Hospital, Moorfields, and to King's College Hospital, London.

The invention of the Ophthalmoscope by Helmholz, in 1851, marks one of the most salient points in the whole history of Ophthalmic Surgery. Before that time, there were no means of ascertaining with precision the condition of the fundus of the living eye; the blackness of the pupil seemed to baffie all attempts to pierce it, and, cataract excepted, it was only in the advanced stages of a few diseases, such as cancer and sub-retinal dropsy, where the growth or floating tunic had approached the lens that a shrewd guess could be made.

The luminosity of the eyes of the carnivora and ruminants must have often engaged the attention of reflecting minds in all ages, and the red glow of the pupil of the human albino could not have altogether escaped notice; but it remained for later times to show that the fundus of the normal human eye, naturally shines with nearly as bright a reflection as that of the lower animals, to determine the conditions under which this phenomenon may always be observed, and to turn this knowledge to a practical account in the detection of disease. The honor of having made the first step towards this, belongs to Mr. Cumming, who communicated to the Royal Medical and Chirurgical Socie ty, June, 1846, a very valuable memoir, embodying the results of a series of well-contrived experiments and observations by which he demonstrated that the fundus of the healthy hnman eye naturally furnishes a brilliant reflection; he also laid down the conditions necessary for seeing it; and in the modifications of its colour and intensity he sought a means of distinguishing the different diseases of the choroid and retina. His labor did not, however, at the time, receive the attention they deserved, and were soon forgotten.

Erlach, a German professor, who wore concave spectacles, unconsciously made a very near approach to the discovery of the ophthalmoscope when he noticed that a friend's eye shone brightly at the moment that his friend saw the image of a lamp reflected in his, the professor's spectacles; here the elements of the ophthalmoscope were accidently present. Until a very recent date it was commonly taught that the blackness of the human pupil depended on the complete absorption by the choroid of the incident rays of light, for which purpose its dark pigment seemed to be a peculiar provision. But this explanation is now known to be incorrect, the apparent blacknesa of the pupil is a necessary result of the optical construction of the eyeball; for when the eye is fixed upon any luminous point, the rays emanating from it, and traversing the pupil, are collected by the lens, and other transparent media to a focus on the retina, where a corresponding image is formed; after which most of the incident rays are thrown back along the same paths by which they entered, and return to the point whence they first started. But since some dispersion takes place, a few of the returning rays will reach the eye of an observer, whenever he occupies a position near the direct line that connects the source of light and the eye examined, in which case its pupil will appear to emit a reddish glow, and this is more vivid when the eye is not properly accommodated, because the dispersion is then greater. Now, under ordinary circumstances the observer does not occupy such a position with reference to the source of illumination and the eye observed, and therefore the returning rays do not meet him, and the pupil consequently appears to him black.

It is obvious that the most favorable position for the observer is one that coincides with the direct line between the light eye under observation. For the simple illumination of the fundus, Cumming's method was succesful, but its practical application to diagnostic purposes was greatly limited, because it did not give images. A few years after the publication of Cumming's paper, Helmholz, by studying the optical construction of the eye with reference to the causes of the apparent colour of the pupil, was led to the invention of the " "*speculum oculi*," or ophthalmoscope, which enabled him easily and conveniently to illuminate the fundus of the eye, and to explore its minutest details. His instrument consisted of a mirror of superimposed oblong slips of glass, packed in a frame, which was fixed at one end of a short blackened copper tube, at an angle of about 30° to the axis of the tube, the other end of which had a contrivance for holding a concave lens. This end the observer applied to his own eye; a lamp was so placed that its rays fell at a convenient angle upon the mirror which threw them back upon the fundus of the eye under the examination, where they underwent a second reflection, and returned to the mirror, this being transparent some rays passed through it, and traversing the axis of the tube and the concave lens, formed an image visible to the eye of the observer. It would be impossible in this place to notice all the modifications which the intrument has undergone. That which I prefer, and am in the habit of using is Liebreich's, which is a small, circular, slightly concave speculum, mounted on a handle, and pierced centrally with a much smaller hole than that generally made in the glass mirrors. Being metal, an accidental fall does not break it, and the smallness of the hole diminishes to a minium the amount of central shadow in the illumination, that results from the absence of the reflecting surface from the centre of the speculum. A clip for holding a small convex or concave lens is hinged to the frame of the speculum, and folds against its back. To larger convex lenses, of two and two and and a-half inches focal lengths, are usually supplied with this ophthalmoscope, and the whole is packed in a strong portable case which is conveniently carried in the breast pocket of a coat. The source of illumination must be steady; a moderator lamp answers extremely well, but the flame of a candle always flickers so much, that it should only be used when nothing else is at hand. Gas is very convenient, the best arrangement that I have seen is one in use at Moorfields; it is an Argand burner with very fine apertures, and has a piece of fine wire gauze fitted to the bottom, which subdivides the draught into a great number of small currents and makes it very uniform. A short chimney is preferable; a tall one produces too rapid a draught. The burner is fitted to a double jointed arm, which can be raised or lowered, and moved from side to side. The room having been darkened (a thick, lined, green baize blind, sufficiently excludes day light), the patient should be seated on a low chair by the lamp, which ought to be near to the side of his head, and on a level with his eye, which must be screened from its direct rays by a small, blackened tin shield fixed to the arm of the burner; or the same object may be gained by moving the lamp backwards, so that his face is in the shade. The observer standing or sitting before his patient receives the rays from the lamp, upon the ophthalmoscope applied to his own eye which looks through the central hole, and by a slight manœuvre throws them upon the patient's eye, the pupil of which at once shines with a bright rew glow. The cornea and lens should be examined before exploring the fundus; beginners often mistake opacities in these structures for those in the vitreous humour and *vice versa*, and it requires some practice to estimate the depth at which opacities are situated. It is well to ascertain previously by oblique illumination, whether corneal nebulæ exist, as by so doing one source of error may be avoided. The ophthalmoscope is of great use in diagnosing the early stage of conical cornea, one side of the cone being illuminated whilst the opposite side is in darkness; it is also of the greatest service in detecting commencing cataract, lenticular opacities, which would otherwise escape detection, appearing as black lines and marks upon red background. The lens and cornea should be examined with a less intense light than that used in exploring the deeper parts; over illumination should always be avoided ; it is quit possible to miss seeing faint striæ in the lens when the light is too intense.

In some cases much information will be gained by ophthalmoscopically examining the iris; the edge of the pupil may be adherent to the capsule of the lens, or fringed with false membranes; perhaps a wound of the cornea has been received, there is a mark upon the iris, and it is uncertain whether a foreign body has passed through it and lodged behind. I recorded a case of this kind, in the Ophthalmic Hospital Reports No. 6, which I saw through the kindness of Mr. Bowman, where, without the ophthalmoscope, it would have been impossible to have ascertained its true nature. An eye was struck by a chip of iron from a chisel. It became slightly inflamed, but got well in a week. A month after the accident the sight became misty.—At this time a small scar was detected near the outer edge of the cornea, and a minute speck of uncertain nature was seen upon the outer part of the iris: it might have been a foreign body, or an ecchymosis, or uvea, or a complete perforation; the ophthalmoscope showed that it was a hole. When the pupil had been dilated with atropine the superficial parts of the lens were found slightly opaque, and near the posterior surface, below the nucleus, a small piece of iron was plainly visible. For a complete examination of the crystalline lens, and thorough exploration of the fundus, the pupil must have been previously dilated with atropine; but where a mere glance at the entrance of the optic nerve and adjacent region is sufficient, a practised observer can obtain this through the undilated pupil and for several reasons, which affect private practice especially, it is better to abstain from the use of atropine, when we can do without it. The disfiguremnnt caused by the great size of one pupil, and the inconvenience produced by the abeyance of accommodation are often complained of.

To examine the choroid or retina, the observer must either approach the patient's eye very closely, in which case he obtains a highly magnified erect view of the fundus; or he holds a bi-convex lens of about two and-a-half inches focal length before it, resting a finger on the patient's forehead to keep the lens steady.

Holding the lens in this manner by a slight to and fro movement of his own head, he hits off the exact distance at which the image of the patient's fundus is formed; at this moment the confused red glow is replaced by a distinct view of the vessels, etc.

The color of the fundus varies greatly, and a knowledge of its modifications cannot be too soon acquired. They mainly depend on the varying proportions of blood and pigment in the choroid. Where the pigment is sparing, as it generally is in fair persons, and the chori-capillaries is naturally full, the color is a bright orange.red; an excess of blood renders the red more decided; a larger quantity of pigment produces a duller or brick-red, which becomes brownish when the pigment is very abundant, and the chorio-capillaries anæmic.

When the pigment is very plentiful the surface of the choroid seems to be mapped out in small brown islands separated by lighter yellowish bands indicating the arrangement of the larger vessels. This appearance is due to the greater abundance of the stellate pigment cells of the stroma in the interspaces between the vessels, than upon them. It was Cumming's belief, that the choroidal pigment reflected more highly than the other tissues in the fundus; but this is not the case, for the sclerotic reflects more light than all the other structures conjointly. The sclerotic reflection is a brilliant white, with a slightly yellowish tinge: in transmission through the choroid it acquires a reddish hue, subject to the modifications I have described.

The extreme tenuity and transparence of the retina render it a bad reflector, so that the membrane itself is not usually seen except by an experienced observer, who detects it as a faint haze overlying the choroid; it is best seen in dark eyes, where the abundant choroidal pigment moderates the splendor of the sclerotic reflection, and prevents it overpowering the fainter retinal hue. The larger retinal vessels, however, yield beautifully distinct images. The entrance of the optic nerve is a very conspicuous object, and it comes into view when the patient slightly converges his eyes. The old term "*papilla*" is inappropriate, because it does not project from the inner surface of the fundus, as was formerly imagined, except very slightly at its margin; centrally it's slightly depressed; its outline is more or less circular; slight deviations are compatible with health, the most common is an elipse with its long axis vertical; an oval or elliptical shape with the long axis horizontal is moro rare, and I have never met with it except in disease. The optic-nerve-entrance, as I shall call it, has a pinkish white color; a paucity of blood in its capillary net makes it pale white; and when atrophied it has a glistening pearly bluish tint. An excess of blood reddens it, and in extreme hyperæmia its color so nearly resembles that of the adjacent fundus, that its situation is best found by tracing the trunks of the large retinal veins to their confluence. Generally, the arteria centralis pierces the optic-nerve-entrance more centrally than the vein; it appears on its inner surface either as a short single trunk that divides at once into an upper and a lower branch, each of which immediately subdivides dichotomously, in such a manner that a couple of large arterial branches take an upward and other two a downward course towards the ora serrata; or very frequently the artery subdivides within the optic-nerve, in which case several large branches appear simultaneously at the inner surface of the entrance. The arteries have a straighter course than their venæ comites from which they are also distinguished by their paler color and smaller calibre. The large venous trunks often make their exit separately, and unite subsequently within the optic-nerve. In addition to the large primary branches several small twigs of the retinal artery radiate in various directions from the optic-nerve-entrance, with the reservation that none run outwards across the "yellow spot," which is supplied by capillaries only, the large branches also passing above and below so as to embrace it. The surface of the optic-nerve-entrance undergoes very important changes in disease, the chief of which is cupping. This is indicated by u bluish white color, and by a sudden diminution of the calibre and an abrupt change of the direction of the venous trunks at its margin. Cupping results from excessive intraocular pressure and atrophy.

Under certain abnormal conditions a spontaneous pulsation is seen in the retinal vessels, the visible movements of the blood being limited to the area of the optic-nerve-entrance, or but slightly overstepping it. This pulsation is generally associated with excessive tension of the globe, of which it is a sign; it may be produced artificially by pr essure with the finger. A slight degree of pressure will evoke the venous pulse; it is a rythmical movement of the blood in the vessel, which is emptied backwards against the course of the current; the swell and collapse of the vein alternate with those of the artery. A heavier pressure produces the arterial pulse, which is synchronous with that at the wrist: and a still greater pressure, arrests all pulsation in the vein and artery, and blanches the entrance of the nerve. When the patient looks straight at the ophthalmoscope the "yellow spot" comes into view. In most eyes this part is characterized by a slight difference of color, which is, however, readily overlooked by inexperienced observers; it generally has a deeper tint than the neighboring parts.

The hyaloid canal, with its obliterated artery, is only very exceptionally seen. I have twice observed a vestige of them in the form of a fine thread stretching forward from the optic-nerve-entrance towards the lens.—*London Med. Review.*

[To be continued.]

